# Human Papilloma Virus as a Possible Factor in the Pathogenesis of Oral Lichen Planus

**Published:** 2009

**Authors:** Sayed Mohammad Razavi, Parichehr Ghalayani, Mohammad Reza Salehi, Hajar Attarzadeh, Mahdi Shahmoradi

**Affiliations:** * Associate Professor, Oral Pathology Department and Torabinejad Dental Research center, Faculty of Dentistry, Isfahan University of Medical Sciences, Isfahan, Iran; ** Associate Professor, Oral Medicine Department and Torabinejad Dental Research center, Faculty of Dentistry, Isfahan University of Medical Sciences, Isfahan, Iran; *** Assistant Professor, Oral Medicine Department and Torabinejad Dental Research center, Faculty of Dentistry, Isfahan University of Medical Sciences, Isfahan, Iran; **** Post Graduate Student, Pediatric Dentistry Department, Faculty of Dentistry, Isfahan University of Medical Sciences, Isfahan, Iran; ***** Dentist, Faculty of Dentistry, Isfahan University of Medical Sciences, Isfahan, Iran

**Keywords:** Human papillomavirus 18, Lichen Planus, Oral, Polymerase chain reaction

## Abstract

**Background:**

Oral lichen planus (OLP) is a common chronic inflammatory mucocutaneous disease. Clinical diagnosis of OLP requires clinical work-up and histologic examination to rule out possible dysplasia and carcinoma. It is possible that oral mucosal viral infections including HPV infection may have a causative role in OLP pathogenesis. The aim of this study was to examine the coincidence of human papilloma virus type 18 and oral lichen planus.

**Methods:**

This study was a case-control study. Twenty nine paraffinized specimens of previously diagnosed oral lichen planus and 14 paraffinized specimens of nonpathogenic mucosa were studied. Polymerase Chain Reaction (PCR) analyze used for detection of DNA HPV 18. The data were analyzed with SPSS software and Fisher’s exact test was used to find the possible relation between HPV18 infection and oral lichen planus.

**Results:**

Nine out of 29 (31.0%) lichen planus samples and one out of 14 (7.1%) controls were HPV 18 positive. No significant correlation (P = 0.128) was observed between HPV18 infection and oral lichen planus.

**Conclusion:**

According to the findings there might be a co-incidence of human papilloma virus type 18 and oral lichen planus.

## Introduction

Oral lichen planus (OLP) is a chronic immunologic inflammatory mucocutaneous disease.^1^ Because of high prevalence of the disease,^2^ painful and irritating nature of erosive types and high potential for malignancy and squamous cell carcinoma formation in some types of OLP, human papilloma virus (HPV) 16 & 18 have posed a great importance to this disease.^1^ HPV 18 is a high risk type of papilloma virus.^3^ According to Campisi^4^, HPV18 was the most prevalent genotype seen in OLP and leukoplakia. In Giovannelli^5^ study, HPV18 was the most prevalent genotype among the oral malignant and premalignant lesions. The etiology of lichen planus involves the degeneration of the basal cell layer of the epithelium induced by cell mediated immunologic reactions. Speculated causative factors such as stress, diabetes, hepatitis-C, trauma, and hypersensitivity to drugs and metals, have different degrees of support.^2^ Recently, viruses like HPV and human herpes virus (HHV) have been found to play a role in the pathogenesis of OLP. Existing data suggest that in OLP, auto-cytotoxic CD8+ T-cells are activated and replace the apoptotic keratinocytes. In fact, CD8+ T-cells cause the apoptosis of virally infected cells. This indicates that oral mucosal viral infections may play a role in the pathogenesis of OLP.^2^,^6^ HPV is a member of papilloma viridea family, with no envelope and with a diameter of 50-500 nm.^3^ Different types of HPV are distinguished based on the degree of homology of sequence of nucleic acid. Some types of HPV have been found to have an association with specific types of premalignant and malignant lesions. HPV infection is strongly associated with dysplasia and cancer of uterine cervix. More than 95% of cervical cancers posses DNA of high risk HPV types like 16, 18 and 31.^3^ Although there are established correlation between different types of oncogenic HPV and some malignant and premalignant diseases,^4^,^7^,^8^ there is no consensus about the relation between the presence of HPV and OLP lesions. In a study by Vesper et al in 1997, predictive value of HPV infection in recognizing OLP was evaluated. In this study HPV 16, 18 and 31 genomes were detected in 42% of OLP cases.^7^ In 2003, Oflatharta et al detected the HPV 16 genome in 26.3% of OLP cases. No HPV genome was detected in controls. Based on the observation, a significant relation between HPV 16 and OLP was proposed.^8^ However, in a study by Campisi et al 4, 19.7% of OLP cases were HPV positive and no relation was found between virus presence and clinical findings. Given the previous studies, it seems that much more researches should be performed to confirm the relation between HPV and OLP. So, the aim of our study was to ascertain if the HPV18 acts as a possible factor in OLP pathogenesis.

## Materials and Methods

**Figure 1:**
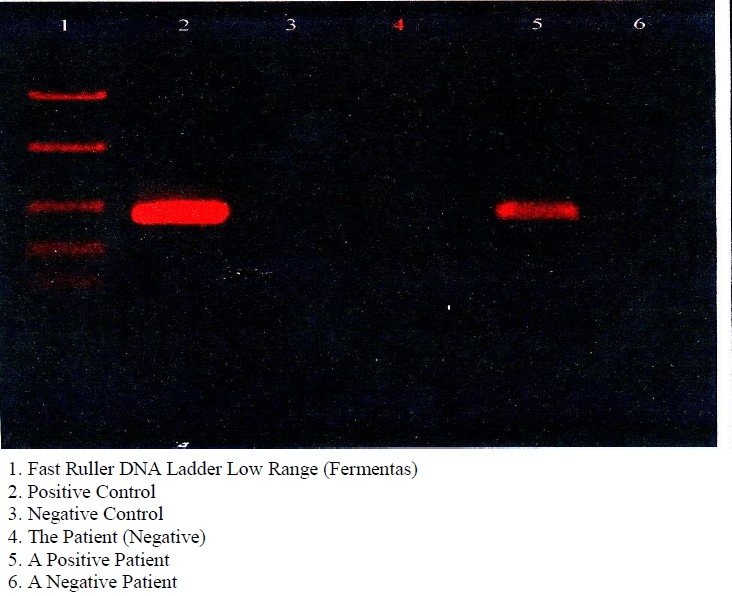
Electrophoresis scan.

This study was a case-control study. The study sample included 29 paraffinized specimens of previously diagnosed oral lichen planus and 14 paraffinized specimens of nonpathogenic mucosa, archived in Oral pathology department, Isfahan University of Medical Sciences between 2002 and 2007. Each paraffin block was assigned a code number and sections were prepared from each block. Inclusion and exclusion criteria were enough tissue in biopsy for HE staining and PCR plus confirmation of diagnosis by two pathologists; the selected cases had only OLP and they had no other diseases. Sections were prepared from paraffin block in the following way: a microtome was placed under a safety hood and was cleaned with xylella and ethylene to remove any debris or pollution. The microtome cutting edge was thoroughly cleaned with xylella and ethylene too. The pliers were disposable and the gloves were renewned after every sample preparation. The sections were prepared consecutively so that the sections with the thickness of 4 microns were used for H-E microscopy and the sections with the thickness of 10 microns were used for PCR. Each section for PCR was placed in a 5 ml tube; 43 tubes were prepared for PCR. Three steps were performed for DNA extraction from paraffin blocks: deparaffinization (to remove the paraffin accompanying tissue),^9^ cell lyses (to destruct the cellular structure for DNA extraction)and DNA purification. Different methods have been used to purify DNA including phenol/chloroform method, simple boiling method, chelex-100 method and using a DNA extraction kit. In this study, we used chelex-100 method to obtain 100 ng of purified DNA.^10^,^11^ After determining the DNA concentration by UV spectrophotometer at 260 nm, and testing the quality of DNA isolation by β-actin amplification in all samples, the PCR technique with a standard consensus primer set GP5+(5’-TTTGTTACTGTGGTAGATACTAC-3’), was used to test for the presence of HPV DNA. The condition used for primer set was as follows: 1.5 mM MgCl_2_, 100 μM deoxynucleotide triphosphate (dNTP), 200 pmoles of primer and 2.5 U of *Taq* polymerase (invitrogen). A total reaction of 50 μl containing 1 μl template DNA was amplified by incubating at 94°C for 5 min followed by 40 cycles (95°C for 60 s, 40°C for 2 min and 72°C for 90s) and 72°C for 10 min. After PCR reaction, the electrophoresis was performed on PCR products and the bands were observed using a UV eliminating Transilluminator (Figure 1). Those subjects that had bands in 2% agarose gel in correct positions in regard to the marker and the control positions were detected as HPV 18 infected subjects. A positive control and a negative control were used along with each sample as PCR controls.^12^ The data were analyzed with SPSS software version 14.0 (SPSS, Inc., Chicago, IL) and Fisher’s Exact test was used to find the possible relation between HPV 18 infection and oral lichen planus.

## Results

**Figure 2:**
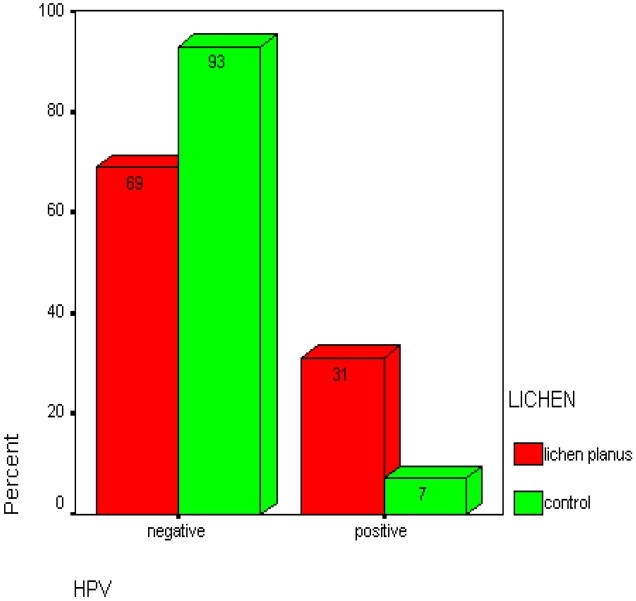
Relative Prevalence of HPV18 in different groups (by percent).

This study included 29 OLP cases with minimum age of 22 years and maximum age of 84 years (mean age of 49.55 ± 13.12) and 14 healthy con-trols with minimum age of 29 and maximum age of 70 years (mean age of 48.5 ± 10.18); 12 of cas-es and 7 of controls were female. Nine out of 29 (31.0%) lichen planus cases and one out of 14 (7.1%) controls were HPV 18 positive (Table 1 & Figure 2). Given the P value gained by Fisher’s exact test (P = 0/128) no significant relation was observed between HPV18 infection and oral lichen planus (Table 2). No significant relation was observed between sex and OLP too (Table 3).

**Table 1:** Relative Prevalence of HPV18 in two groups.

HPV18	negative	positive	Overall

Group			
Cases	20	9	29
controls	13	1	14
all	33	10	43

**Table 2:** Relative Prevalence of cases based on the site of lesion.

Site	Tongue	Lip mucosa	Gingiva	Floor of mouth	Buccal mocosa	Overall

Group							
Case	3 (%10.3)	1 (%3.4)	1 (%3.4)	2 (%6.9)	22 (%75.9)	29 (%100)
Control	1 (%7.1)	1 (%7.1)	4 (%28.6)	0 (%0)	8 (%57.1)	14 (%100)
All	4 (%9.3)	2 (%4.7)	5 (%11.6)	2 (%4.7)	30 (%69.8)	43 (%100)

**Table 3:** Relative Prevalence of HPV18 based on sex.

	HPV18	Positive	Negative	Overall	P value

SEX					
Male	Lichen plan	4	8	12	0.245
	Control	0	7	7	
	Overall	4	25	29	
Female	Lichen plan	5	12	17	0.629
	Control	1	6	7	
	Overall	6	18	24	

## Discussion

A lot of researchers have found that racial and geographical features may affect viral factors which involve in human diseases. Some viral factors have been studied and proposed as etiologic factors in oral lichen planus. Researchers showed that CD8+ cells cause apoptosis in virally infected cells. So it may be possible to consider a role for viral infections in OLP pathogenesis.^6^ Molecular and epidemiologic studies suggest that HPV infection in upper respiratory tract may play a role in the pathogenesis of head and neck tumors.^12^,^13^ Also the role of HPV in premalignant lesions has been studied.^5^,^14^–^15^ In 2000, Sand et al 14 found the HPV genome in 27.3% of OLP lesions. In 2002, Oswald et al reported the presence of HPV 16 and 18 in 9.4% of OLP cases.^15^ In 2006, Giovannelli et al reported the presence of HPV 16, 18, 33 and 35 in 22.4% of cases.^5^ In the present study, HPV 18 genome was found in 31% of OLP lesions and 7.1 % of controls. In 2009, Szarka^16^ reported that HPVs may be involved in the development or progression of potentially malignant oral lesions including oral lichen planus. Increased risk of HPV infection was reported in separate studies by Campisi^4^, Giovannelli^5^ and Furrer et al 17.

However in the present study, no significant difference was observed between the case and the control groups regarding HPV 18 infection. In 2006, Giovannelli reported that HPV infection can be affected by keratinization, so that keratinized tissue is more resistant to HPV infection.^5^ Increased rate of proliferation in non-keratinized tissue can make it more susceptible to HPV infection. In the present study, HPV genome was found in 88.88% of non-keratinized tissues (lip mucosa, mucosa of the floor of mouth and buccal mucosa) and in 11.11% of keratinized tissues (tongue mucosa and gingiva)). Given the P value of Fishers exact test in this study (near the significant level), an increase in the number of study subjects may lead to a significant difference between case and control groups regarding the HPV 18 infection. Possibly, finding the effect of HPV 18 in OLP lesions needs a larger study sample. Also, there was no significant relation between HPV 18 infection and the site of lesion. Given the P value in male group (0.245) and in female group (0.629), no significant relation was observed based on sex in OLP group.

## Conclusion

In the current study, no significant relation was observed between HPV /18 infection and OLP. According to the findings, there might be a coincidence of human papilloma virus type 18 and oral lichen planus. Given the fact that HPV 16 is a high risk type, it is suggested that another study be conducted to investigate the relation between HPV16 and other subtypes of HPV and OLP lesions.

## References

[1] Greenberg MS, Glick M (2003). Burket’s Oral Medicine: Diagnosis and Treatment. Philadelphia: B.

[2] Neville B, Damm DD, Allen CM, Bouquot J, Ne-ville BW (2001). Oral & Maxillofacial Pathology.

[3] Braunwald E, Fauci AS, Kasper DL, Hauser SL, Longo DL, Jameson JL (2001). Harrison’s Principles of Internal Medicine.

[4] Campisi G, Giovannelli L, Arico P, Lama A, Di Liberto C, Ammatuna P (2004). HPV DNA in clinically different variants of oral leukoplakia and lichen planus. Oral Surg Oral Med Oral Pathol Oral Radiol Endod.

[5] Giovannelli L, Campisi G, Colella G, Capra G, Di Liberto C, Caleca MP (2006). Brushing of oral mucosa for diagnosis of HPV infection in patients with potentially malignant and malignant oral lesions. Mol Diagn Ther.

[6] Sugerman PB, Savage NW (2002). Oral lichen planus: causes, diagnosis and management. Aust Dent J.

[7] Boyd AS, Annarella M, Rapini RP, Adler-Storthz K, Duvic M (1996). False-positive polymerase chain reaction results for human papillomavirus in lichen planus. Potential laboratory pitfalls of this procedure. J Am Acad Dermatol.

[8] OFlatharta C, Flint SR, Toner M, Butler D, Mabruk MJ (2003). Investigation into a possible association be-tween oral lichen planus, the human herpesviruses, and the human papillomaviruses. Mol Diagn.

[9] Banerjee SK, Makdisi WF, Weston AP, Mitchell SM, Campbell DR (1995). Microwave-based DNA extraction from paraffin-embedded tissue for PCR amplification. Biotechniques.

[10] Atzei D, Ferri T, Sadun C, Sangiorgio P, Caminiti R (2001). Structural characterization of complexes between iminodiacetate blocked on styrene-divinylbenzene matrix (Chelex 100 resin) and Fe(III), Cr(III), and Zn(II) in solid phase by energy-dispersive X-ray diffraction. J Am Chem Soc.

[11] Soto Y, Valdes C, Mune M, Pimentel T, Ramirez R (1998). Detection of type 16 human papillomavirus DNA in formalin-fixed invasive squamous cells from laryngeal cancers by polymerase chain reaction. Mem Inst Oswaldo Cruz.

[12] Tyring SK (2000). Human papillomavirus infections: epidemiology, pathogenesis, and host immune response. J Am Acad Dermatol.

[13] McGhee EM, Cotter PD, Weier JF, Berline JW, Turner MA, Gormley M et al (2006). Molecular cytogenetic characterization of human papillomavirus16-transformed foreskin keratinocyte cell line 16-MT. Cancer Genet Cytogenet.

[14] Sand L, Jalouli J, Larsson PA, Hirsch JM (2000). Human papilloma viruses in oral lesions. Anticancer Res.

[15] Ostwald C, Rutsatz K, Schweder J, Schmidt W, Gundlach K, Barten M (2003). Human papillomavirus 6/11, 16 and 18 in oral carcinomas and benign oral lesions. Med Microbiol Immunol.

[16] Szarka K, Tar I, Feher E, Gall T, Kis A, Toth ED (2009). Progressive increase of human papillomavirus carriage rates in potentially malignant and malignant oral disorders with increasing malignant potential. Oral Microbiol Immunol.

[17] Furrer VE, Benitez MB, Furnes M, Lanfranchi HE, Modesti NM (2006). Biopsy vs. superficial scraping: detection of human papillomavirus 6, 11, 16, and 18 in potentially malignant and malignant oral lesions. J Oral Pathol Med.

